# Novel Three-Dimensional and Biocompatible Lift-Off Method for Selective Metallization of a Scleral Contact Lens Electrode for Biopotential Detection

**DOI:** 10.3389/fmedt.2022.920384

**Published:** 2022-06-10

**Authors:** Sven Schumayer, Nicolai Simon, Benjamin Sittkus, Sandra Wagner, Volker Bucher, Torsten Strasser

**Affiliations:** ^1^Institute of Microsystems Technology, Furtwangen University, Furtwangen, Germany; ^2^Institute for Ophthalmic Research, University of Tuebingen, Tuebingen, Germany; ^3^IMTEK—Department of Microsystems Engineering, University of Freiburg, Freiburg, Germany; ^4^University Eye Hospital Tuebingen, Tuebingen, Germany

**Keywords:** three-dimensional direct writing, lift-off technology, gelling sugar, contact lens electrode, biocompatibility, electrode configuration

## Abstract

Presbyopia describes the eye's physiological loss of the ability to see close objects clearly. The adaptation to different viewing distances, termed accommodation, is achieved by a change in the curvature of the eye lens induced by the ciliary muscle. A possible approach to correct presbyopia could be to detect the ciliary muscle's neuromuscular signals during accommodation and transfer these signals electronically to a biomimetic, micro-optical system to provide the necessary refractive power. As a preliminary step toward such a described system, a novel three-dimensional and biocompatible lift-off method was developed. In addition, the influence of the distance between the electrically conducting surfaces of the lens on the accommodated signal amplitudes was investigated. Compared to the conventional masking methods, this process has the advantage that three-dimensional surfaces can be masked with biocompatible gelling sugar by utilizing a direct writing process with a dispensing robot. Since gelling sugar can be used at room temperature and is water-soluble, the process presented is suitable for materials that should not be exposed to organic solvents or excessively high temperatures. Apart from investigating the shrinkage behavior of the gelling sugar during the physical vapor deposition (PVD) coating process, this paper also describes the approaches used to partially coat a commercial scleral contact lens with an electrically conductive material. It was shown that gelling sugar withstands the conditions during the PVD processes and a successful lift-off was performed. To investigate the influence of the spacing between the electrically conductive regions of the contact lens on the measured signals, three simplified electrode configurations with different distances were fabricated using a 3D printer. By testing these in an experimental setup, it could be demonstrated that the distance between the conductive surfaces has a significant influence on the amplitude. Regarding the described lift-off process using gelling sugar, it was found that the dispensing flow rate has a direct influence on the line uniformity. Future work should address the influence of the viscosity of the gelling sugar as well as the diameter of the cannula. It is assumed that they are the prevailing limitations for the lateral resolution.

## Introduction

Accommodation describes the change of refractive power of the eye's crystalline lens to receive a sharp image of objects at different viewing distances (1). Over human life, this ability fades gradually until finally, at the age of about 65, only depth of focus remains. This process is called presbyopia and becomes noticeable around the age of 40 years (2). The reasons for presbyopia are still being discussed today but can possibly be attributed to the physiological decrease in lens plasticity (3). Uncorrected presbyopia already leads to a worldwide productivity loss of about 11 billion US-Dollars at a working age of 50 years. Assuming a working age of 65 in the future, the global productivity loss would increase even further to 25.4 billion US-Dollars. This is caused by the difficulties people experience while doing general tasks requiring near vision such as reading, writing, and driving (4). It is expected that presbyopia will affect around 2.1 billion people worldwide in 2030 (5). Summarizing these facts together, it is plausible that further approaches to correct presbyopia should be developed.

Scleral contact lenses belong to the group of therapeutic contact lenses designed to protect or assist in the healing process of areas of the ocular surface consisting of the cornea, sclera, and associated epithelial layers (6). A scleral lens is used here because it has an increased diameter of up to 25 mm compared to conventional contact lenses (7). It thereby spans the entire cornea, which is the most sensitive part of the anterior eye, and only touches the eye in the area of the scleral conjunctiva (8). In the past, scleral contact lenses have also been applied as a carrier for electrodes to detect the neuromuscular signals of the ciliary muscle during accommodation (9–13). These so-called biopotentials are induced by cellular activities that directly lead to a shift in extracellular potential. The signals measured in this way are of great importance in diagnostics, as they can provide information about abnormalities of muscles, nerves, or the brain via the signal characteristics (14). As one example, contact lens electrodes are used for recording the electroretinogram (ERG) to provide diagnostic information about the retina (15). Even though contact lens electrodes for ERGs are already in practical application, there is no commercially available scleral contact lens electrode for detection of ciliary muscle signals. The reliable detection and further processing of the ciliary muscle's signals could give a better understanding of the accommodation process and lead to possible new corrective approaches.

To detect electrical potentials of the ciliary muscle during accommodation at the ocular surface with a commercially available contact lens, certain areas of the polymer lens must be made electrically conductive. The required segmentation can be achieved either by a subtractive method like etching or an additive lift-off procedure (16). However, etchants used in the subtractive method could harm the substrate (17). In the lift-off process, the required segmentation is achieved by masking the areas which should not be coated before depositing the electrically conductive material (16–19). After deposition, the masking material can be dissolved and the unwanted metal is “lifted off” to reveal the patterned conductive surfaces (16–19). The photoresists commonly used for this purpose are removed by organic solvents (e.g., acetone) after metal coating (20). Consequently, such processes are not compatible with chemically sensitive contact lens materials such as fluorosilicone methacrylate copolymer. These polymers, being tailored to the physiology of the eye, are highly sensitive to the organic solvents, and any contact with, for example, acetone, isopropanol, or ethanol would lead to the loss of the lens transparency. Instead of a photosensitive coating, alternative materials for sacrificial layers such as gelatin (21), agar-agar (21, 22), shellac (23), paraffin wax (24), sugar (25, 26), or even ice (27) are available. These are already widely used to manufacture different microstructures, for instance, brain implants (26) or various microfluidic systems (22, 24, 27). Because of their biocompatibility, gelatin (28–31), agarose (29, 30, 32–35), and sugar (33, 34) are widely used for tissue engineering such as matrix materials for bioprinting. The first two are used as carrier materials in hydrogel-based bioinks containing the living cells and act as a type of scaffold to support the mechanical integrity of the printed parts (29, 31). There are several techniques for bioprinting, with the main applications being inkjet, extrusion, or laser-assisted printing (31). Compared to conventionally utilized materials in a lift-off process, sugar has positive aspects such as environmental friendliness and cost efficiency (25). Thus, a two-dimensional lift-off process was already carried out using maltose (25). Utilizing commercially available 3D printing filament and an extrusion 3D printer, it was additionally demonstrated that a lift-off process is possible on three-dimensional structures in order to produce, for instance, three-dimensional, selectively metallized high-frequency circuits (36). In this work, a three-axis dispensing robot was combined with the biocompatible and room temperature printable sacrificial material, gelling sugar. The gelling sugar, which withstands the conditions during the metallization process, was successfully used to mask a scleral contact lens in three dimensions and to perform a lift-off after metallization. Unlike agar-agar, which has a melting point between 51 and 54°C (22), or gelatin, which turns into a solution above 35°C (30), preliminary tests showed that gelling sugar retains its shape even at a temperature of 100°C. This temperature stability, together with the fact that no organic solvents are required due to the water solubility, makes gelling sugar more advantageous compared to paraffin wax, with a melting point of 65°C (24) or shellac, with a glass transition temperature of 43°C (23). Since gelling sugar is a food product, it can be assumed that any residues are biocompatible and would not affect the physiology of the eye.

## Materials and Methods

### Determination of the Printing Parameters

#### Masking

**Figure 1:**
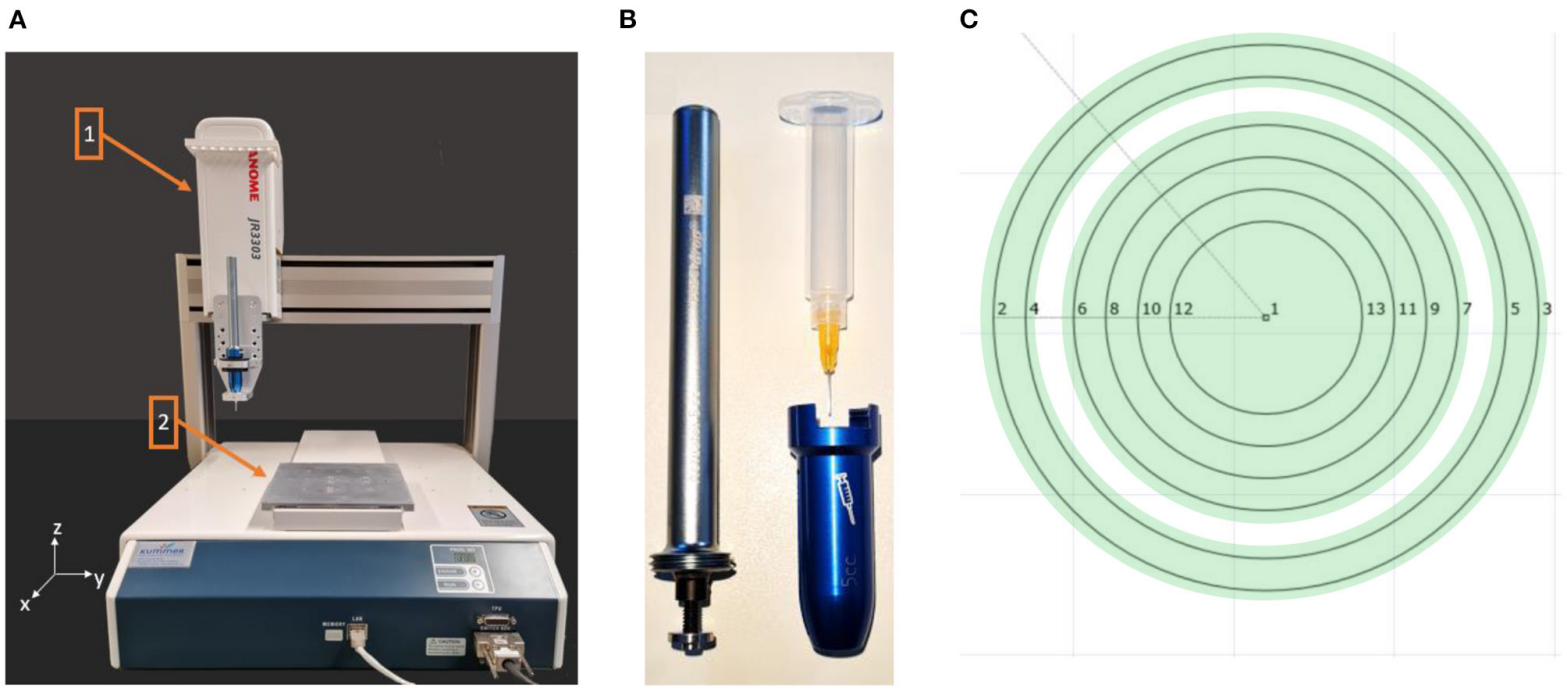
**(A)** The upper part (1) of the dispensing robot moves in y- and z-direction while the positioning table (2) moves in x-direction. **(B)** The dosing unit consists of the volumetric dispenser with the cartridge and a dosing cannula. **(C)** The stepwise travel path of the dispensing unit defined in the JR C-Point II V8 software for masking the surfaces (green) of the lens, which should not be coated.

To prepare the high-viscosity gelling sugar, the manufacturer's recommended ratio of 10 g of granulated gelling sugar (2 plus 1, Suedzucker AG, Mannheim, Germany) was mixed with 10 ml of distilled water in a 100 ml beaker and then boiled on a hot plate (US150, Stuart, England) for 2 min. Before the solution could be dispensed by the dispensing robot (Figure 1A), it was filled in a 5 cc dispenser cartridge using a 10 ml disposable syringe. Manual filling of the cartridge with the disposable syringe was intended to avoid air inclusions, as this allowed the gelling sugar to be distributed in the cartridge in a more controlled manner. Any air inclusions would lead to an inconsistent volume flow, resulting in interrupted dispensing or reduced linewidth. A dosing cannula (JP Kummer Semiconductor Technology GmbH, Augsburg, Germany) with an inner diameter of 0.33 mm was attached to the cartridge before it was inserted into the volumetric dispenser (Dispenser PreciFluid, France), as shown in Figure 1B. The dispensing unit was then secured in the holder of the dispensing robot (JR3303, Janome, Hachioji, Japan). For determining the printing parameters, a flat 1.5 mm thick polycarbonate (PC) substrate was used.

Before substrate was attached to the positioning table with Kapton, the PC was plasma-activated to increase the surface energy. For this purpose, the material was first cleaned with distilled water and dried under a nitrogen flow. The substrate was manually activated in a serpentine-like movement, maintaining a minimum distance of 3.0 mm between the substrate and the tip of the plasma activation unit (kINPen Neoplascontrol, Germany). Subsequently, the distance between the dispensing needle and the attached PC substrate was calibrated to 0.3 mm. The dispensing-test program for the dispensing robot was written using the JR C-Point II V8 software (Janome, Hachioji, Japan) and a volume flow of 1.1 μl/s was set on the dispensing control unit (Dispenser PreciFluid, France). As part of the test program, three rows consisting of eight lines, each 15 mm long, were dispensed at different feed rates (2.5–20 mm/s) at a room temperature of 20°C. The gelling sugar was then dried for 60 min in a climatic chamber (Binder MKF 115 E1.3, Germany) at 80°C.

#### Measuring

To determine the shrinkage behavior of the gelling sugar during the coating process, the sample was measured before and after metallization. For this purpose, the linewidths of the different feed rates were measured at three different locations, each using a two-point measurement of the digital microscope (VHX-700F, Keyence, Osaka, Japan) at a magnification factor of 50.

#### Coating

The titanium-gold layer was deposited using an evaporation and sputtering system (AUTO 306, HHV Ltd, England). The base pressure (evacuation level before processing) was set in the range of 2–4 × 10^−6^ mbar. First, the surface was plasma cleaned with a glow discharge. For this purpose, an argon plasma was ignited at 3 kV at 50 mA and applied for 7 min at a chamber pressure of 8 × 10^−2^ mbar. This was followed by sputtering of the titanium adhesion promoter layer at 3 × 10^−3^ mbar and a plasma power of 100 W. To keep the pressure constant, a needle valve was used for manual readjustment. After deposition of the 20 nm thick titanium layer (deposition rate of 0.95 Å/s), the needle valve was closed until the base pressure was reached again. Then the evaporation process of the gold started. This was done by thermal evaporation with a boat current of about 140 A. To keep the radiant heat exposure on the contact lens low during the boat heating process, the power was ramped up with the shutter closed. To increase homogeneity, the target was rotated throughout the coating process and the film thickness was controlled *in situ* with a calibrated quartz crystal. The gold deposition was stopped at a thickness of 150 nm (deposition rate of 7.5 Å/s).

The theoretically expected electrical resistance (R) was calculated considering the specific gold resistance (ρ), the respective average circumference of the individual electrode (l), and their expected cross-sectional areas (A), as shown in the following equation.


(1) $$\begin{array}{r} R=\rho \frac{l}{A}\left[\Omega \right] \end{array}$$

For the inner conductive area, a mean diameter of 14.5 mm and a cross-sectional area of 1.5 × 10^−4^ mm^2^ were assumed, while for the outer conductive area, a mean diameter of 18.5 mm and a cross-sectional area of 2.25 × 10^−4^ mm^2^ were supposed. These underlying assumptions were made concerning the feasibility of the manufacturing process. Thus, the electrical resistance of 6.7 Ω was calculated for the inner electrode surface and a resistance of 5.7 Ω for the outer electrode surface.

#### Post-processing

During post-processing, the PC substrate was immersed in an ultrasonic bath (RK 100H, Bandelin, Germany) containing distilled water. This dissolved the gelling sugar and the test structures remained.

### Testing the Electrode Configuration

**Figure 2:**
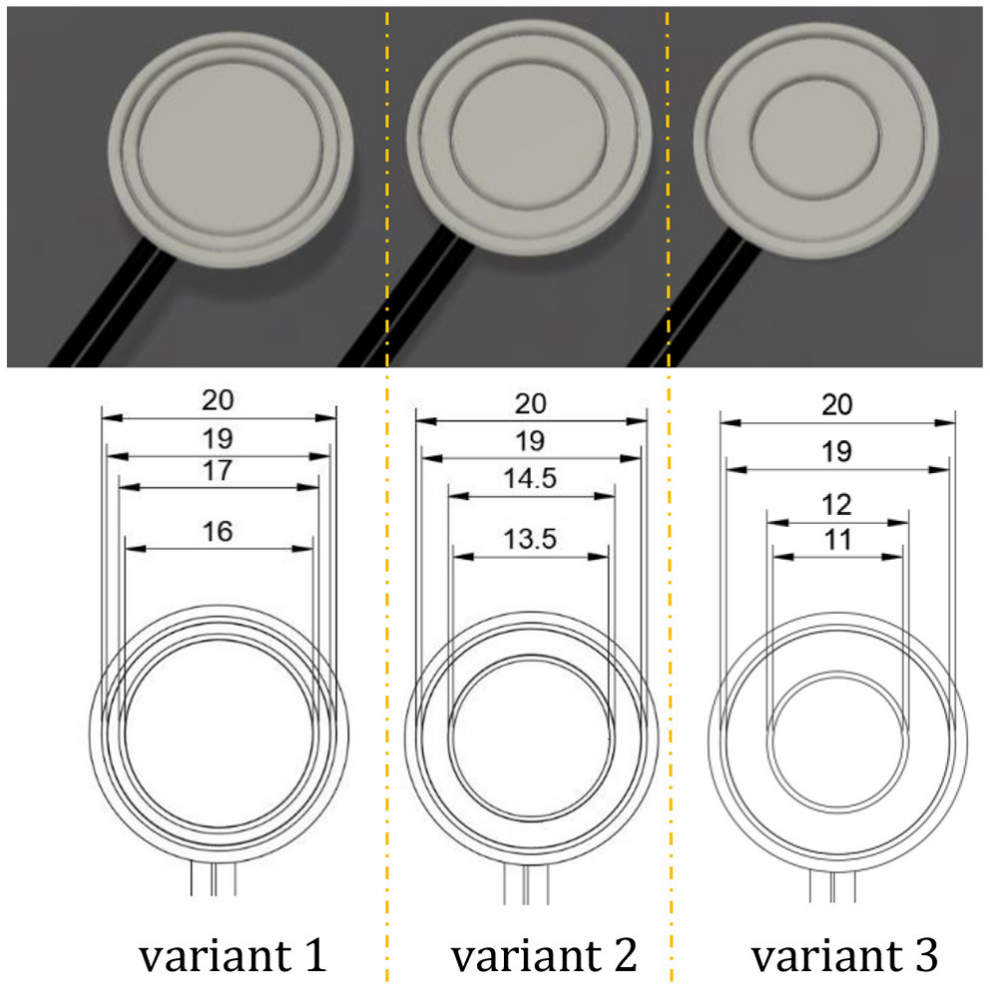
Variant 1 is an electrode configuration with a reduced distance between the two conductors, while variant 2 is the ideal arrangement that considers the physiology of the ciliary muscle. Variant 3 is an electrode configuration with a maximum spacing of the detecting electrodes. A greater distance could result in the inner electrode interfering with the user's field of view in low light conditions.

Literature values for neuromuscular voltages emitted by the ciliary muscle and detected by the contact lens electrodes range from few micro- to millivolts (9–13). Hence, it appeals to optimize the contact lens electrode so that it detects as large amplitude values as possible. For this purpose, the influence of the distance between the two concentric conductive surfaces of the lens electrode on the amplitude level was investigated. For the fabrication of the test electrodes, circular holders with different distances of concentric guide rails were printed using the 3D printing FDM method. A conductor wire (switch wire YV; 1.1 mm diameter; Conrad Electronics, Berlin, Germany), stripped to the appropriate length, was then inserted into the provided holes of the holders along the guide rails, starting from the backside. A rendering of the resulting test electrodes and their dimensions can be seen in the following CAD illustration in Figure 2.

**Figure 3:**
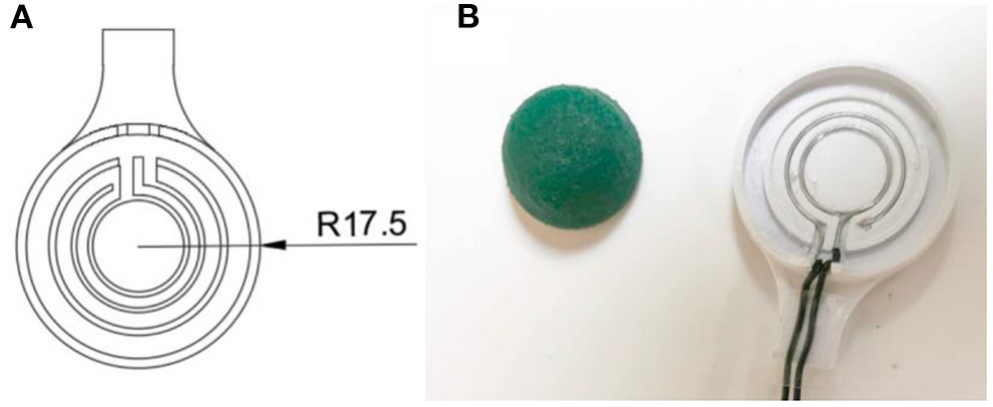
**(A)** The left side shows a simplified technical drawing of the electrode plate, while the right side **(B)** shows the wired plate next to the soaked foam.

For testing, an experimental setup consisting of a frequency generator (4080 Function Generator, PeakTech, Ahrensburg, Germany), an electrical voltage emitting base plate, and an oscilloscope (TDS 210, Tektronix, Beaverton, OR, United States) was arranged. The baseplate shown in Figure 3 was 3D-printed via FDM and stripped conductor wires were added in the same manner as the fabricated testing electrodes. The spacing of the corresponding concentrically emitting conductor wires considers the muscle physiology of the ciliary muscle. Within the first 2.0 mm posterior to the scleral spur, the greatest change in ciliary muscle thickness occurs during contraction and decreases laterally (37). Hence, one assumption is that the contraction gradient is accompanied by the electrical sum potential of the ciliary muscle. The scleral spur running concentrically around the lens axis has an average distance of about 6.0 mm to the axis [cf. (38)]. Thus, the average radius of the inner conducting wire is 7.0 mm. The radius for the outer conductor wire was set to 9.5 mm. These electrode diameters were also considered when determining the diameter of the electrode variant 2 (Figure 2) as well as for the finally fabricated contact lens electrode.

A 10.0 mm thick synthetic resin foam soaked in a saline solution was placed on the emitting electrodes to simulate the contact resistance of the eye, which is mainly dominated by the impedance of the cornea [Z = 8–16 kΩcm^2^ (39)].

The voltage measurements with the three detecting electrode configurations were performed on the described experimental setup. A voltmeter was used to set a square wave voltage at the transmitting electrodes with the high level in the range of 3.9–4.7 V, a low level at 0 V and a frequency of 0.5 Hz to be able to measure signals in the expected millivolt range at the test electrodes (12). Each of the electrode configurations was measured in two measurement series with 10 repetitions. Between the series of measurements, the resin foam was soaked again in the saturated saline solution and the square wave voltage was recalibrated.

### Manufacturing of the Contact Lens Electrode

#### Contacting and Masking

**Figure 4:**
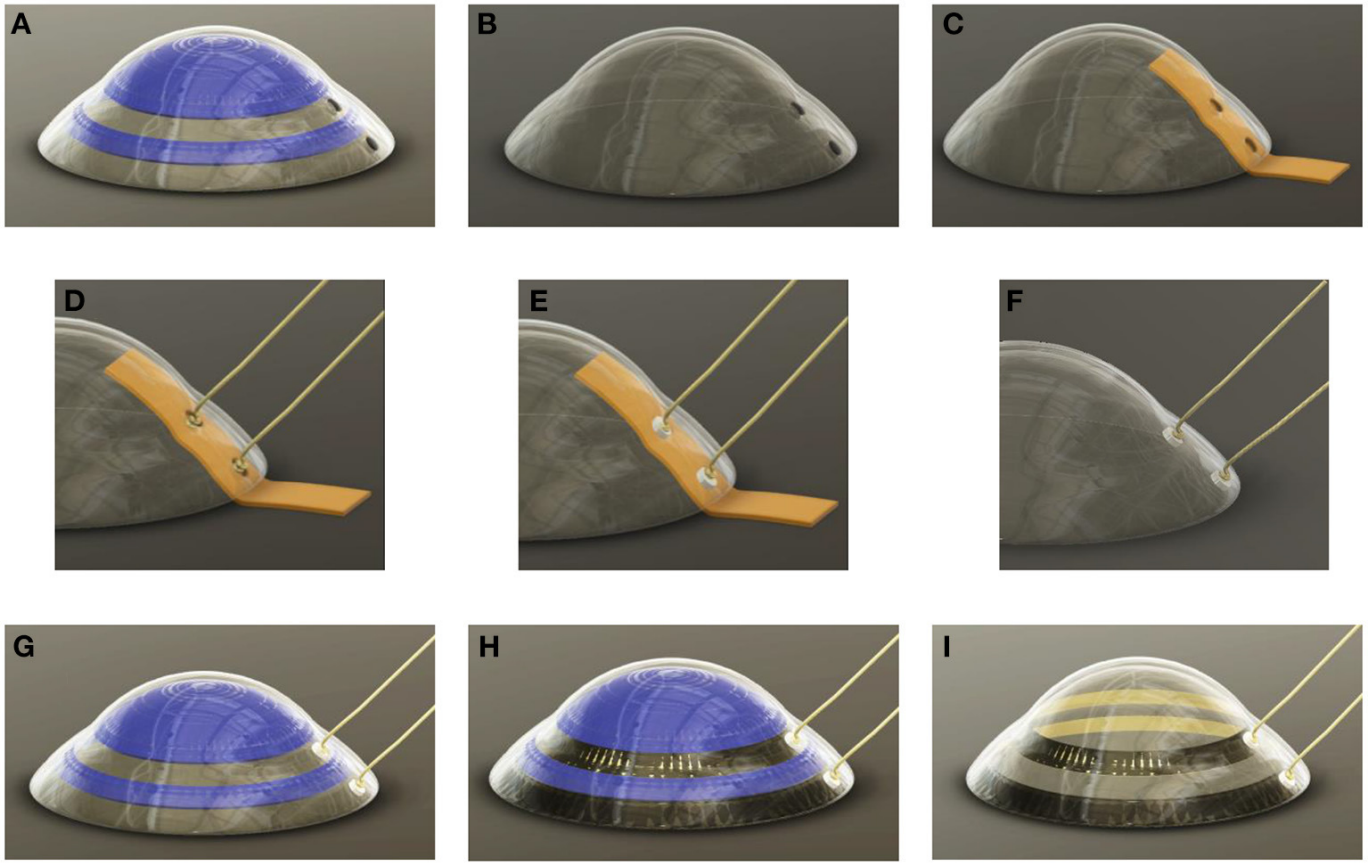
An illustration of the individual work steps. **(A)** The gelling sugar (blue) is used as a drilling template **(B)** and can then be dissolved in water. **(C)** The concave side is then covered with Kapton® tape (orange) to protect the lens from adhesive flowing in. **(D)** The centering of the gold wires in the middle of the drill holes can then take place **(E)** before the drilling holes are filled with the biocompatible and electrically conductive adhesive. **(F)** After the adhesive has cured, the Kapton® can be peeled off **(G)** and the concave side of the lens can be masked a second time with gelling sugar, leaving the electrode sites unmasked. **(H)** This is followed by the metal coating of the lens. For this purpose, titanium is first sputtered as an adhesion promoter before a gold layer is evaporated directly on it without vacuum breakage. **(I)** Afterwards, the gelling sugar mask is removed with water as solvent.

The high-viscosity gelling sugar, used to mask the 20 mm contact lens (ASF-120, Woehlk Contactlinsen GmbH, Schönkirchen, Germany), was prepared in the same manner as described in “Masking” section. Due to the lens geometry with different radii, a cannula (JP Kummer Semiconductor Technology GmbH, Augsburg, Germany) with a larger diameter of 0.61 mm was used to reduce the travel distance for masking different lens segments. Prior to the insertion of the scleral contact lens into a custom-developed 3D printed holder on the positioning table of the dispensing robot, the lens substrate was prepared. Thereby, the lens was first rinsed with distilled water, wiped dry with a cleaning cloth, and finally, blown off with nitrogen. To increase the wettability of the lens polymer, a plasma activation was performed in the same procedure as the test substrate. Then, an offset between the dispensing cannula and the lens which was fixed in a 3D-printed holder was set to 0.3 mm. The program for the dispensing robot was written using the JR C-Point II V8 software (Janome, Hachioji, Japan) and the defined areas (Figure 1C) were then masked with gelling sugar at a flow rate of 10 mm/s and a volume flow of 4 μl/s set on the dispensing control unit. Afterwards, the gelling sugar was dried in a climatic chamber at 80°C for 20 min. This first application of the gelling sugar served to define the connection points of the contacts of the later deposited electrically conductive surfaces holes. The individual work steps are schematically shown in Figure 4. Two holes, each with a diameter of 1.0 mm, were drilled (Micromot TBM 220, Proxxon, Hetzerath, Germany) on the convex lens surface for electrical contact before the gelling sugar was subsequently washed off. The drill holes on the concave side were taped with Kapton tape. The ends of the two insulated gold wires, about 10 cm long and 0.1 mm thick, were first stripped with a lighter flame, then twisted around a 0.8 mm diameter needle and positioned in the drilling holes. The positioned wire ends were then glued into the drill holes using a conductive biocompatible adhesive (EPO-TEK MED H20S, Epoxy Technology Inc, Billerica, MA, USA). A handheld dispenser (THE-200; TAEHA Corporation, Namyangju-Si, Korea) set to a pressure of 351 kPa and a time of 0.024 s was used for this purpose.

After curing the conductive adhesive, the Kapton tape was removed. This was followed by further plasma activation of the concave lens side and re-masking with gelling sugar. The masked contact lens was then dried for 3 h in a climate chamber (MKF 115 E1.3, Binder GmbH, Tuttlingen, Germany) at 80°C and <5% humidity. After drying, the masked scleral lens was coated with a biocompatible metal film consisting of titanium as the adhesive layer and gold as the electrode material.

#### Coating

The coating was done in the same procedure as described in detail in “Coating” section.

#### Post-processing

**Figure 5:**
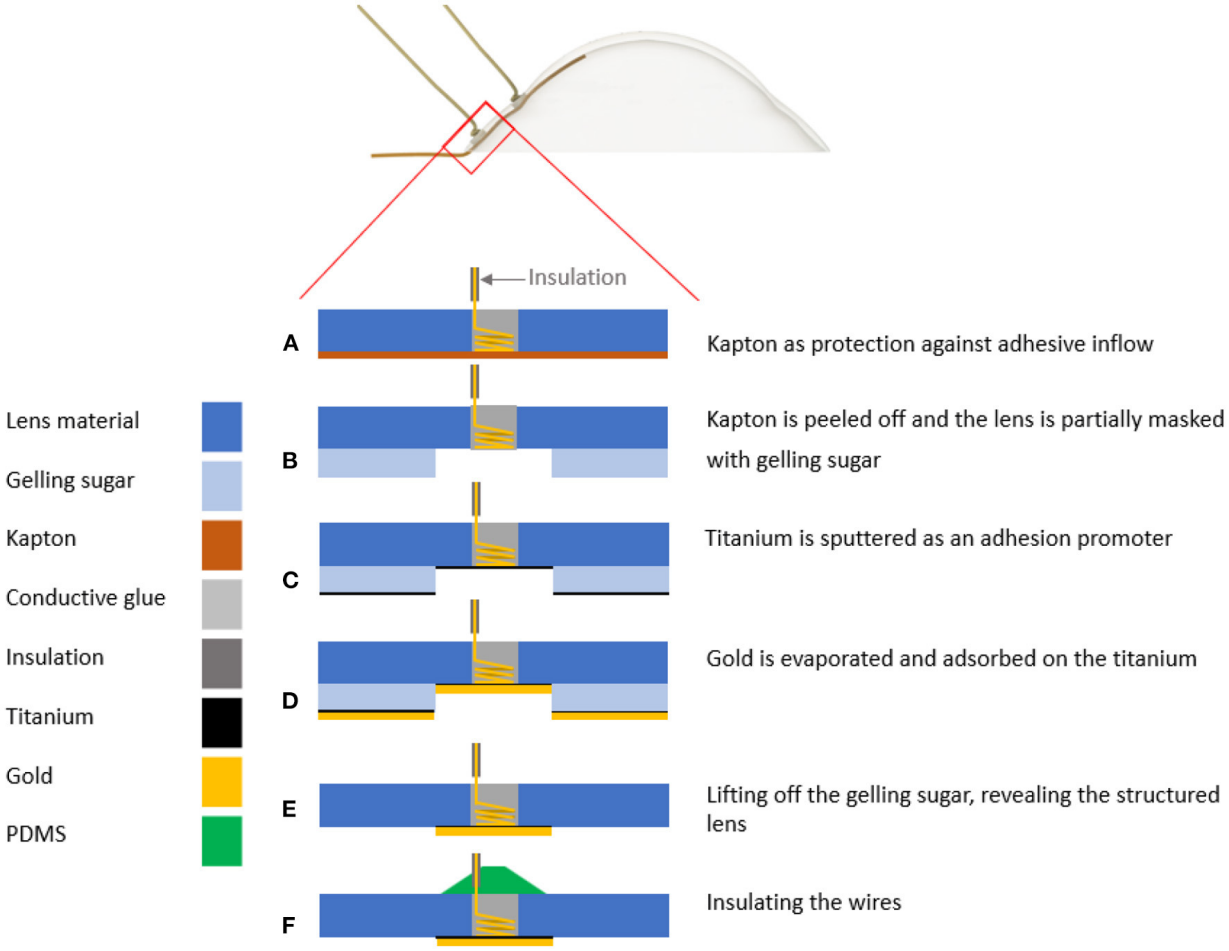
Schematic representation of the steps of the lift-off procedure. **(A)** After the biocompatible, conductive adhesive has cured in the drill hole, the Kapton® can be peeled off. **(B)** Masking of the substrate on the concave side with gelling sugar is performed **(C)**, followed by sputtering of the titanium layer with a thickness of 20 nm. **(D)** Immediately afterwards, the gold layer with a thickness of 150 nm is deposited. **(E)** The lift-off step follows, in which the gelling sugar is dissolved with distilled water in an ultrasonic bath. **(F)** Finally, the contact points of the convex side of the lens are insulated with silicone polydimethylsiloxane (PDMS).

After the coating procedure (Figures 5A–D), the sample was immersed in an ultrasonic bath with distilled water. While the gelling sugar dissolved in the water bath, the partially coated scleral contact lens electrode was revealed (Figure 5E). The exposed contacts on the convex side of the lens were then sealed with PDMS (SILPURAN 4200; Wacker Chemie AG, Munich, Germany) (Figure 5F). The PDMS applied in this way had to cure for 30 min on a hot plate (US150; Stuart, England) at 80°C. To establish a connection between the lens electrode and a measuring unit, two DIN connectors (1.5 mm) had to be soldered on at 220°C in a further step. The two solder joints were then insulated with heat-shrink tubing.

## Results

### Printing Parameter

**Figure 6:**
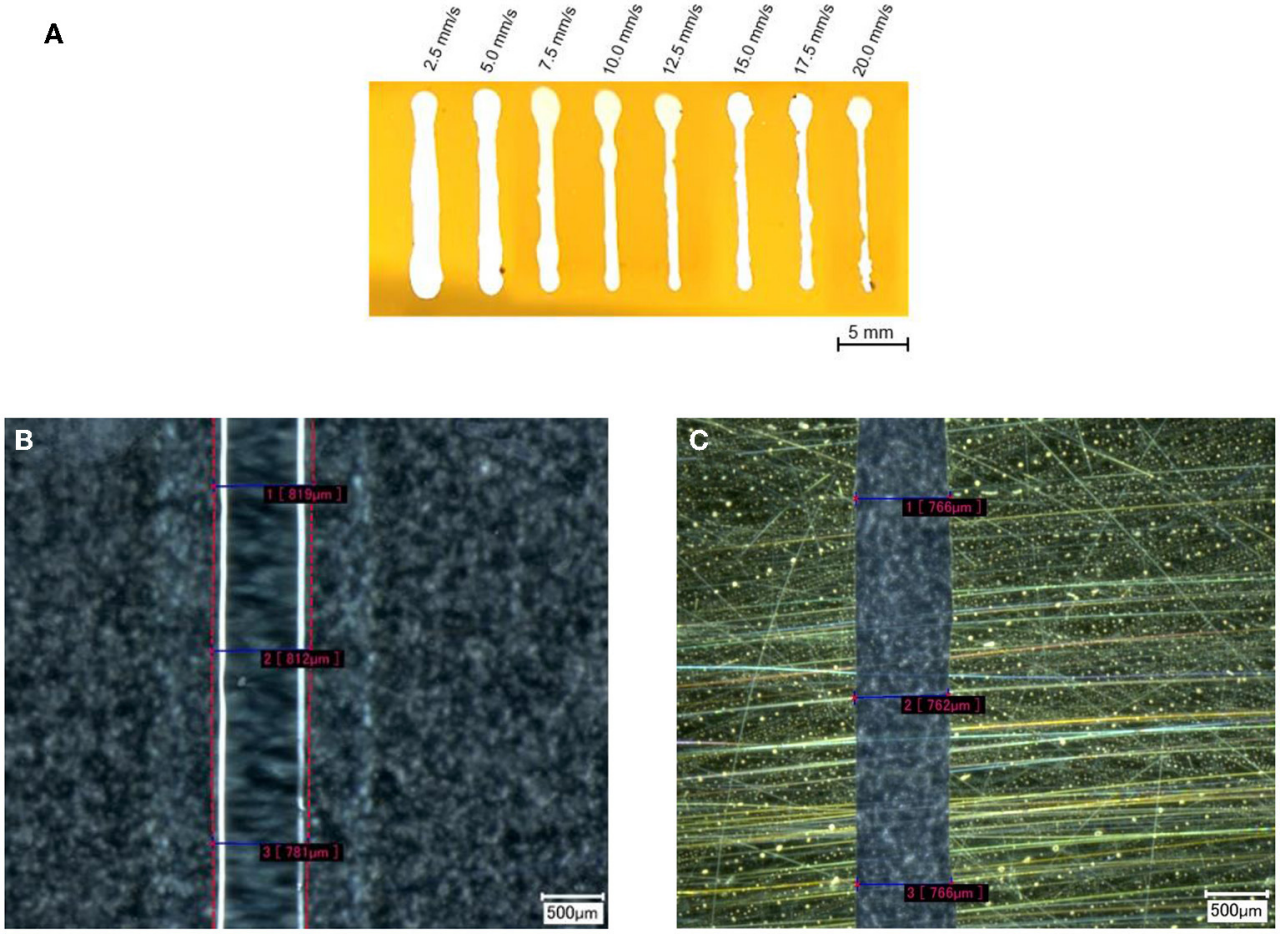
**(A)** Image of the coated test substrate with different feed rates after the lift-off process. The different feed rates are written above the lines. The image was post-processed by increasing the brightness to get a better edge contrast. **(B)** The measured line width at a feed rate of 12.5 mm/s, before the metallization and the same line after **(C)** the lift-off. The pink dashed lines in **(B)** were post-processed to visualize the edge of the gelling sugar.

To accurately mask the contact lens, the possible dissolution and shrinkage behavior must be considered. Figure 6A shows the coated PC-test substrate, demonstrating that droplet-like masking occurs at the beginning of dispensing and that the correct linewidth becomes visible only as dispensing progresses. As expected, a thinner linewidth can be achieved by keeping the volume flow constant and increasing the feed rate. The lines with a feed rate between 10 and 15 mm/s show the highest uniformity, while linewidths above this range become narrower, but edge fidelity decreases. This could also be observed in the other two-test series.

**Figure 7:**
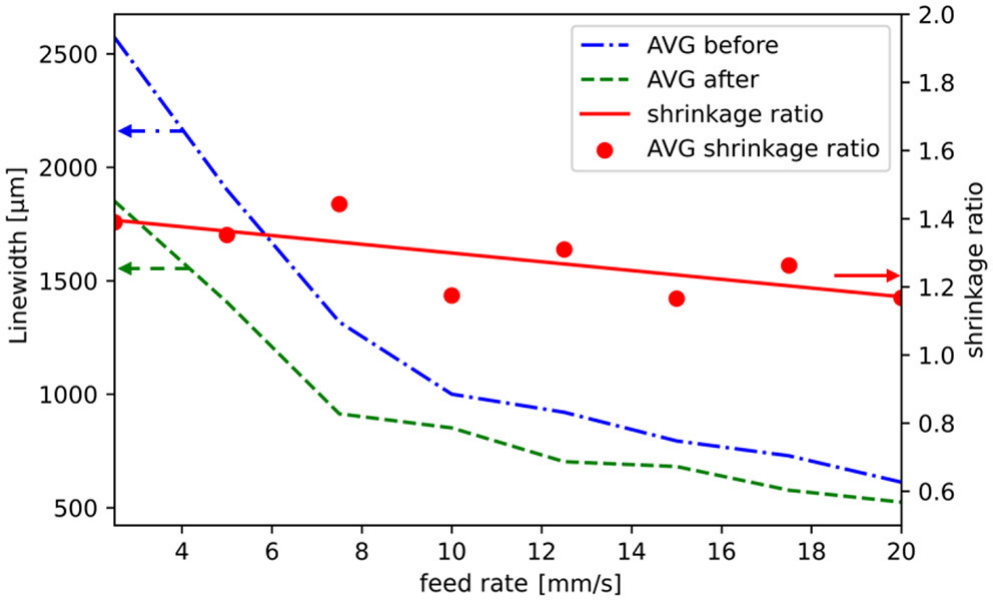
Averaged values (AVG) which had been measured before (blue dash-dot line) and after (green dash line) the metallization of the test substrate. The average shrinkage ratios of the different feed rates are represented as red dots, whereas the linear regression of the shrinkage ratio is represented by the red line. The shrinkage ratio decreases with higher feed rates. Note that the linewidth axis starts at 500 μm and the shrinkage ratio axis starts at 0.6. The arrows present the affiliation of the graphs to the axes.

Figure 6B shows the PC substrate masked at 12.5 mm/s, and Figure 6C shows the same line after the coating and lift-off process. While the edge fidelity is high at this feed rate, significantly poorer edge fidelity values were obtained at lower feed rates (2.5–7.5 mm/s) as well as at higher feed rates (17.5–20.0 mm/s) (cf. Figure 6A). The calculated shrinkage rate (linewidth before/linewidth after) of 1.05 for the structure width shown in Figures 6B,C is even below the range of the average shrinkage rate of 1.28 (SD = 0.101), as depicted in Figure 7.

### Electrode Configuration

**Figure 8:**
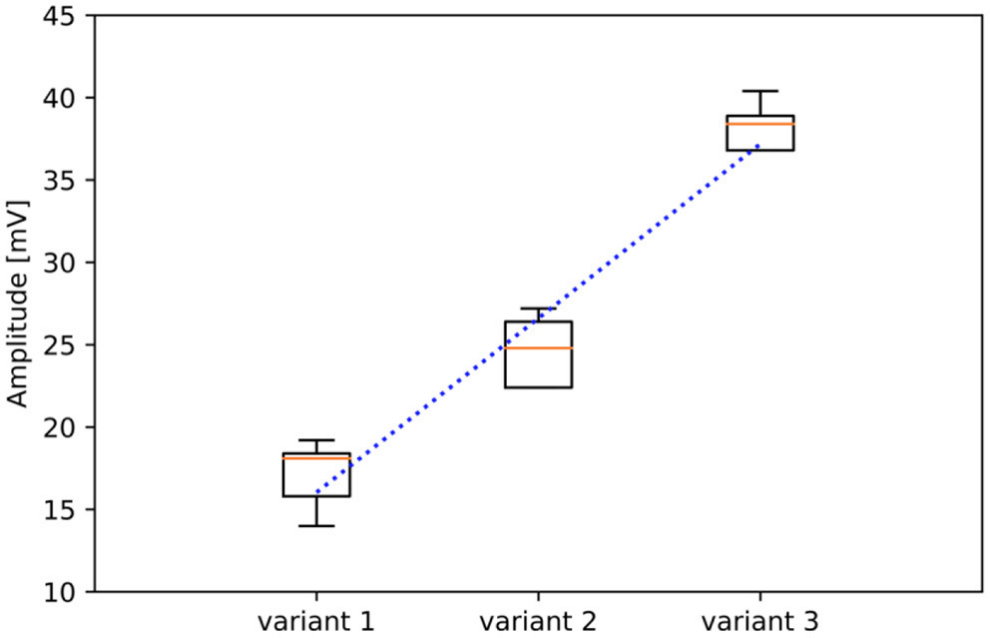
Linear correlation (blue dot line) between the measured voltage amplitude and the different electrode variants, whose conductive surface distance differs by 1.25 mm in each case. The orange lines show the median of the single measurements, which can be seen with the corresponding boxplot. Each configuration was measured 20 times. Note that the y-axis starts at an amplitude voltage of 10 mV.

The following combination of a line graph and boxplot (Figure 8) shows the relationship between the distance from the inner to the outer conductive surface and the determined voltage amplitude in millivolts, fitted with a linear regression (blue dot line). The orange lines show the median of the twenty individual measurements of the respective configurations. With a reduced distance between the two conductive surfaces (variant 1), the average measured voltage is 18.6 mV, while with the physiologically motivated layout (variant 2) it is 26.3 mV, respectively. With an increased distance between the two conductive surfaces (variant 3) it is 39.0 mV. Based on the physiologically motivated layout, this means that a reduction in the distance of 44.4% between the conductive surfaces results in a reduction of the amplitude level by almost 30%, while an amplitude increment of 50% can be achieved by increasing the distance by 155.5%.

### Manufacturing of the Scleral Contact Lens Electrode

**Figure 9:**
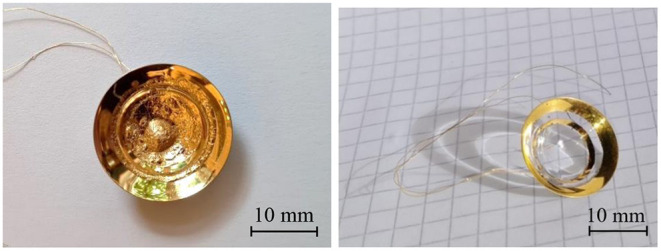
The partially coated scleral contact lens electrode before (left) and after (right) the lift-off procedure.

The gelling sugar was successfully applied selectively to the lens using the robot dispenser. The linewidth could be varied by changing the volume flow and the cannula diameter. Furthermore, Figure 9 shows that the gelling sugar also withstands the coating process (left) and could easily be removed (right) within a minute using water in an ultrasonic bath. Measuring the linewidths unveiled a negligible shrinking behavior of the sugar with the printing parameters used for the lenses. Further, no partial delamination of the conductive surfaces was evident on the lens after the ultrasonic bath. The electrical resistance measured with the two-wire measurement method was about 6 Ω for the inner conducting surface and about 5 Ω for the outer surface.

## Discussion

Here we presented a new 3D masking process and its successful application for partial coating of a scleral contact lens electrode. Areas of the three-dimensional substrate could be masked with gelling sugar and removed with water after the coating process without leaving any residue. This approach was inspired by the contributions of Byford et al., (36) and Zhang et al., (25). Whereas Zhang et al. showed that two-dimensional lift-off is possible using maltose as a water-soluble masking material, Byford et al. demonstrated that three-dimensional printing is capable of creating lift-off masks using commercial 3D printing filament. The conditions prevailing during the PVD processes used, such as temperature and vacuum, showed no effect at the interfaces to the gelling sugar. The electrical resistance of 6 Ω for the inner conducting surface and about 5 Ω for the outer surface is within the range of previous theoretical predictions (cf. “Coating”).

While conventional masking methods require organic solvents to remove the masking layer after the coating process (20), this is not necessary with this newly described method using gelling sugar. In comparison to alternative masking methods using paraffin wax (24), agarose gel (22), or shellac (23), it was demonstrated that gelling sugar exhibited a higher temperature resistance of up to 100°C. Moreover, gelling sugar can be applied at room temperature, which simplifies the equipment needed. The use of a three-axis dispensing robot enables a direct three-dimensional partial masking, without further need of a mask or development steps compared to conventional masking methods (e.g., spin coating, spray coating, or dip coating).

The results of the printing parameter feed rate at a constant volume flow of 1.1 μl/s indicate an ideal range (10–15 mm/s) in which a high uniformity of the structure can be achieved. Feed rates above or below this range show a reduction in uniformity. If the feed rate is too slow, too much gelling sugar is applied to the substrate, resulting in a non-uniform distribution. The resulting backlog causes the gelling sugar to bulge to all sides of the cannula. If the feed rate is too high, not enough gelling sugar is applied to the substrate. The high viscosity of the gelling sugar and the outlet area limited by the inner diameter result in a drop-shaped structure at the start of the dosing process. Until the dispenser robot has reached the new starting point for a dispensing process, excess gelling sugar can accumulate at the tip of the cannula. This can be reduced by various actions: for instance, a retraction can be set in the dispensing unit after each dispensing process. Alternatively, the dispensing needle can also be cleaned manually. When masking the contact lens, no drop shape was seen at the starting point of the dispensing robot. This can be attributed to the fact that closing the dispensing ring homogeneously distributed the gelling sugar. The investigated shrinkage behavior during the line testing suggests, that higher dimensional accuracy is achieved with smaller structure widths respectively at higher feed rates. Possibly, this can be attributed to a disproportionate decrease in volume compared to the gelling sugar surface area.

The analysis of the influence of the distance between the conductive surfaces on the detectable signal amplitude suggests that with increasing distance between the concentric surfaces a larger voltage is detected. The varying high level of the square wave voltage (3.9–4.7 V) within the measurements is probably due to the different saturation of the saline-soaked synthetic resin foam and the associated free ions. The results indicate that increasing the distance between the two conductive surfaces of the contact lens increases the amplitude of the measured biopotentials. This could be realized by reducing the circular conductive ring area, and by considering the positioning of the conductive surfaces with respect to the anatomical changes of the ciliary muscle during accommodation [cf. (37)]. Considering these improvements as well as the measurements on the experimental setup, we expect that the idealized contact lens electrode will measure in the millivolt range. Furthermore, the bipolar electrode arrangement and the differential measurement should minimize artifacts, for example caused by eye movements (10, 12). We could successfully show that even substrates not suitable for high temperature or sensitive for organic solvents can be masked with this new masking method based on gelling sugar. It can be concluded that gelling sugar does not adversely affect the eye because sugar-based biomaterials have the advantage of being biocompatible and due to its temperature resistance, certain edge fidelity can be expected. New findings were obtained which should help improve the process described. For example, new handling equipment will help to simplify the process. Since the holes are drilled by hand, there will be a permanent limitation in terms of accuracy, which will limit the distance between the two conductive surfaces. Still some open questions remain: a too high dispensing feed rate has a negative impact on the uniformity of the structure width, nevertheless, the influence of the viscosity of the gelling sugar and the thinner cannula diameters was not taken into account. Even finer structure widths would be possible if the viscosity of the gelling agent could be reduced, for example by heating during the metering process. Future investigations will focus on this and the production-specific boundary conditions of the maximum permissible process temperatures. These findings could open up further applications for this new masking method, e.g., for the production of brain electrodes or structuring microfluidic systems.

## Data Availability Statement

The raw data supporting the conclusions of this article will be made available by the authors, without undue reservation.

## Author Contributions

SS, SW, and TS contributed to the conception of the different electrode layouts and test-setup. NS and BS contributed by optimizing the PVD process and producing test samples. SS and VB contributed by researching alternative methods and sacrificial layers as well as developing the three-dimensional lift-off. SS contributed by analyzing the data and writing the first draft of the manuscript. SS and SW wrote the introduction and SW proofread the final manuscript. All authors contributed to manuscript revision, read, and approved the submitted version.

## Funding

This project was funded by the Carl Zeiss Foundation Breakthroughs at Universities 2020: Intelligent Solutions for an Aging Society, the University of Tuebingen, the Faculty of Medicine of the University of Tuebingen, and the Center for Ophthalmology at the University of Tuebingen.

## Conflict of Interest

The authors declare that the research was conducted in the absence of any commercial or financial relationships that could be construed as a potential conflict of interest.

## Publisher's Note

All claims expressed in this article are solely those of the authors and do not necessarily represent those of their affiliated organizations, or those of the publisher, the editors and the reviewers. Any product that may be evaluated in this article, or claim that may be made by its manufacturer, is not guaranteed or endorsed by the publisher.
